# Parallel Tests Using Culture, Xpert MTB/RIF, and SAT-TB in Sputum Plus Bronchial Alveolar Lavage Fluid Significantly Increase Diagnostic Performance of Smear-Negative Pulmonary Tuberculosis

**DOI:** 10.3389/fmicb.2018.01107

**Published:** 2018-06-15

**Authors:** Lin Fan, Danfeng Li, Shaojun Zhang, Lan Yao, Xiaohui Hao, Jin Gu, Hong Li, Jinxia Niu, Zhemin Zhang, Changtai Zhu

**Affiliations:** ^1^Clinic and Research Center of Tuberculosis, Shanghai Key Laboratory of Tuberculosis, Shanghai Pulmonary Hospital, Tongji University School of Medicine, Shanghai, China; ^2^Department of Laboratory Medicine, Shanghai Jiao Tong University Affiliated Sixth People’s Hospital, Shanghai, China

**Keywords:** parallel tests, culture, Xpert MTB/RIF, SAT-TB, diagnostic performance, smear-negative, pulmonary tuberculosis

## Abstract

At present, tuberculosis remains a serious threat to human health. The diagnosis of pulmonary tuberculosis (PTB) is still difficult, and the prominent challenge for diagnosis is the lack of a highly sensitive and specific method. In order to explore the diagnostic value of parallel tests, this study prospectively enrolled 258 patients with smear-negative PTB from May 2, 2015 to December 31, 2016. The sputum specimens and bronchial alveolar lavage fluid (BALF) samples from all patients were assessed for MTB detection by culture, Xpert MTB/RIF, and simultaneous amplification and testing method for TB (SAT-TB). Overall, the sensitivity of any single test using culture, Xpert MTB/RIF, or SAT-TB was lower than that for parallel tests (*p* < 0.05), and the sensitivity rates for MTB detection in BALF were significantly higher than those in sputum samples. There were lower agreements in the detection results between sputum samples and BALF for all tests (*p* < 0.05). The parallel tests models of using culture plus Xpert MTB/RIF plus SAT-TB, culture plus Xpert, or culture plus SAT-TB achieved higher sensitivities compared with all three single test models (*p* < 0.05). Additionally, joint detection using sputum and BALF samples achieved a high sensitivity (0.8566, 95% CI: 0.8086–0.8941). In conclusion, the parallel tests model using culture, Xpert MTB/RIF, and SAT-TB in sputum plus BALF significantly increases the diagnostic performance of smear-negative PTB; thus, this method should be applied clinically when PTB is suspected but smear results are negative.

## Introduction

Tuberculosis (TB) remains one of the leading infectious diseases in the world and is a serious detriment to public health. According to the updated global TB report released by the World Health Organization [WHO] (2017), there were an estimated 10.4 million new cases of TB and an estimated 1.3 million TB-related deaths among HIV-negative people (down from 1.7 million in 2000) in 2016, as well as an additional 374,000 deaths among HIV-positive people that year (World Health Organization [WHO], 2017).

The difficulty in TB diagnosis is one of the important contributing factors in the struggle to control TB. The traditional sputum smear and culture method for TB detection is widely used in the clinical laboratory diagnosis of TB, but its sensitivity is low. Two molecular methods, Xpert MTB/RIF and SAT-TB (simultaneous amplification and testing method for TB), have several advantages, but they still fail to diagnose some cases due to the typical use of only a single sample.

Xpert MTB/RIF is a molecular method for the detection of *Mycobacterium tuberculosis* (MTB) that has excellent diagnostic efficacy in terms of assay-time and positive detection rate, and it has been approved by the WHO (Steingart et al., 2013, 2014). Xpert MTB/RIF also yielded high diagnostic specificity for pulmonary tuberculosis (PTB) (Penz et al., 2015; Kaur et al., 2016; Sehgal et al., 2016; Hasan et al., 2017; Li et al., 2017; Lombardi et al., 2017; Rufai et al., 2017). Additionally, Xpert MTB/RIF provides high efficacy MTB detection in bronchial alveolar lavage fluid (BALF) and non-respiratory samples, such as body fluid, fine needle aspiration, and different tissues, and it presents useful diagnostic value for extrapulmonary TB (Barnard et al., 2012; Penz et al., 2015; Sehgal et al., 2016; Rufai et al., 2017).

Simultaneous amplification and testing method for TB is another fast molecular tool for MTB detection (Fan et al., 2014; Yan et al., 2017). Previous studies showed that the specificity of SAT-TB is almost 100% in detecting PTB (Yan et al., 2016a,b), suggesting that SAT-TB has potential clinical value in the diagnosis of PTB as novel assay. Another advantage of SAT-TB tests is that, because their detection target is RNA rather than DNA, they detect live bacteria.

Although both Xpert MTB/RIF and SAT-TB are highly specific tests, when they are used in isolation and with only a single sample as is typical, there is still a significant deficiency in their sensitivities, which causes some cases of TB to be missed. We hypothesized that the use of these high specificity tests in combination to simultaneously detect MTB in sputum and BALF samples may increase the diagnostic sensitivity of PTB. In this study, we applied parallel tests using culture, Xpert MTB/RIF, and SAT-TB to jointly detect MTB in sputum and BALF samples from patients with PTB to assess the value of parallel detection.

## Materials and Methods

### Subjects

From May 2, 2015 to December 31, 2016, 258 patients with PTB were prospectively enrolled into the study from the Shanghai Pulmonary Hospital, Tongji University School of Medicine, Shanghai, China. In this group of patients, the median age was 32.5 years (range: 11–89 years), 140 cases were male, and 118 cases were female.

The inclusion criteria were: PTB patient, aged > 18 years old, HIV-negative, positive BCG vaccination history, negative results from at least two sputum smears at the start of treatment, willing to be examined by bronchoscopy and have both sputum and BALF specimens collected. All patients enrolled in this study signed informed consent forms, and the study was approved by the Institutional Review Board of Shanghai Pulmonary Hospital, Tongji University School of Medicine, Shanghai, China (Approval No. K15-191). The applied PTB diagnostic criteria followed the WHO guidelines for the treatment of TB and were based on a combination of clinical symptoms, chest radiological evidence compatible with active TB, histological observations, lack of improvement in response to a course of broad-spectrum antibiotics (excluding anti-TB drugs, fluoroquinolones, and aminoglycosides), and a decision by the attending clinician that the patient had a satisfactory response to all courses of anti-TB therapy (World Health Organization [WHO], 2010).

The exclusion criteria were smear-positive results from consecutive sputum specimens, HIV-positive, unclear or ambiguous final diagnosis, reluctance to being tested by bronchoscopy, or failure to collect both sputum and BALF specimens.

### Specimen Collection

Once the smear-negative suspected cases of PTB were enrolled, sputum and BALF specimens were both collected directly after the patients had finished being examined via electron bronchoscopy. BALF samples were collected as follows: a volume of 40–60 ml of sterile saline (0.9%) was instilled into the airway of the affected lung segment, and 30 ml of BALF was collected. These samples were then tested for MTB via BACTEC MGIT 960 culture, SAT-TB assay, and Xpert MTB/RIF. The laboratory staff were blinded to the final diagnostic category (based on the PTB diagnostic criteria described above), and the clinicians were blinded to the results of the two molecular detection tests.

### Bacterial Culture, Xpert MTB/RIF, and SAT-TB Assays

Bacterial culture was performed in a BD BACTEC^TM^ MGIT^TM^ 960 Mycobacteria Culture System (Becton Dickinson and Company, Allschwil, Switzerland) according to manufacturer’s instructions.

The Xpert^®^ MTB/RIF assay was performed by following the manufacturer’s protocol (Cepheid, Sunnyvale, CA, United States). Briefly, 500 μl of decontaminated and concentrated sample was pre-treated with a sample solution (containing NaOH and isopropanol) at a 1:3 ratio for 15 min at room temperature and then was poured into a single-use disposable cartridge that was placed into the GeneXpert^TM^ Dx module. This assay produced results in less than 2 h. Each PCR run comprised internal controls for sample processing (DNA extraction) and PCR validity (presence of inhibitors), and these positive and negative controls were tested every day. The system automatically interpreted all results from the measured fluorescent signals, using embedded calculation algorithms, into the following categories: invalid (if PCR inhibitors were detected with amplification failure), negative, or positive. If categorized as positive, the results were further scaled into four additional categories (very low, low, medium, and high) depending on the detected bacterial load [2, 4].

The SAT-TB assay was performed as previously described (Fan et al., 2014). Briefly, MTB 16S rRNA was isolated from each sample and reverse transcribed to generate a 170-bp DNA fragment. The specific MTB 16S rRNA sense primer containing the T7 promoter sequence was 5′-AATTTAATACGACTCA CTATAGGGAGAGTAGGCCGTCACCCCACCAACAAGCTG-3′, and the antisense primer was 5′-CTGGGAAACTGGGTCTAATAC-3′. The probe sequence was 5′-CCAGCCACGGGAUGCAUGCUGG-3′ and was labeled with 6-carboxyfluorescein (FAM) phosphoramidite at the 5′ end and with 4- [4-(dimethylamino)phenylazo] benzoic acid *N*-succinimidylester (DABCYL) at the 3′ end. Real-time PCR was performed in a 7500 real-time PCR system (Applied Biosystems, Inc., Foster City, CA, United States).

### Patient Follow-Up

All patients included in this study were followed for at least 6 months by the Out-patient department. New chest-CT results were examined every 2 months for an efficacy evaluation of the administered chemotherapy.

### Statistical Analysis

According to the principle of parallel test results reporting, the sensitivities of joint tests were recorded and analyzed separately. The collected data were analyzed by SPSS 21.0. The sensitivity proportion is presented with a 95% confidence interval. Sensitivity was calculated as the proportion of the number of positive test results within the group of patients clinically diagnosed with PTB. Specificity should be calculated with the proportion of the numbers of negative result with Non-TB patients. A *Kappa* value was used to assess the agreement of two methods. Constituent ratio difference was examined by chi-square tests, and *p*-values of less than 0.05 were considered statistically significant.

## Results

### Comparisons of the Sensitivities of Culture, Xpert MTB/RIF, and SAT-TB

**FIGURE 1:**
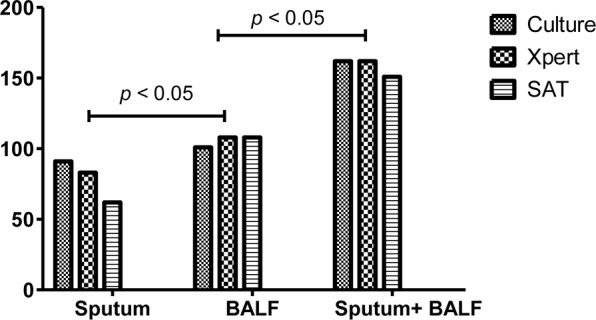
The positive rates of culture, Xpert MB/RIF and SAT-TB in different specimen types. BALF, bronchoalveolar lavage fluid.

**FIGURE 2:**
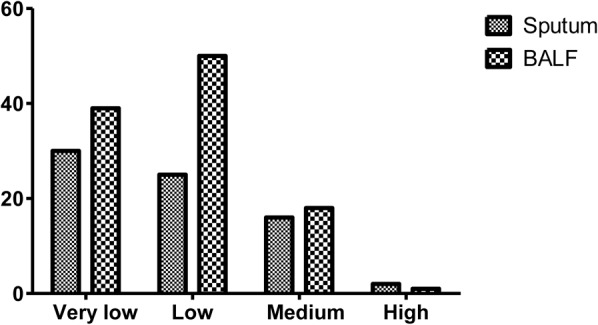
MTB loads according to Xpert MTB/RIF results. BALF, bronchoalveolar lavage fluid.

Three methods were applied to testing the sputum and BALF samples from the 258 included patients with confirmed PTB. The positive rates of culture, Xpert MTB/RIF, and SAT-TB in sputum were 0.3527 (0.2970–0.4128), 0.3217 (0.2677–0.3810), and 0.2403 (0.1922–0.2960), respectively; and those in BALF were 0.3915 (0.3339–0.4522), 0.4186 (0.3600–0.4796), and 0.4186 (0.3600–0.4796), respectively (Supplementary Material 1). Overall, the positive MTB detection rates in BALF were significantly higher than those in sputum samples, and the MTB detection based on joint samples had a higher positive rate than that based on a single sample (*p* < 0.05, **Figure 1** and **Table 1**). The quantitative results from Xpert MTB/RIF indicate that the MTB levels were very low to medium in the majority of patients (**Figure 2**).

**Table 1:** Comparisons of the sensitivities and 95% confidence intervals of each single MTB detection test with different sample types.

	Sputum	BALF	Sputum+BALF
Culture	0.3527 (0.2970–0.4128)	0.3915 (0.3339–0.4522)	0.6279 (0.5675–0.6846)
Xpert	0.3217 (0.2677–0.3810)	0.4186 (0.3600–0.4796)	0.6279 (0.5675–0.6846)
SAT	0.2403 (0.1922–0.2960)	0.4186 (0.3600–0.4796)	0.5853 (0.5243–0.6437)


BALF, bronchoalveolar lavage fluid.

### Agreement Among Culture, Xpert MTB/RIF, and SAT-TB Results for Sputum and BALF Samples

Based on both sputum and BALF samples, the *kappa* values of the test results from culture, Xpert MTB/RIF, and SAT-TB were 0.5, 0.22, and 0.252, respectively (**Tables 2–4**). These relatively low agreement values suggest that there were differences in the detection results between two samples (*p* < 0.05).

**Table 2:** Agreement of culture test results between sputum and BALF samples.

		Sputum	
		Positive	Negative
BALF	Positive	30	71
	Negative	61	96


**Table 3:** Agreement of Xpert MTB/RIF results between sputum and BALF samples.

		Sputum	
		Positive	Negative
BALF	Positive	29	79


	Negative	54	96




*BALF, bronchoalveolar lavage fluid.*

**Table 4:** Agreement of SAT-TB results between sputum and BALF samples.

		Sputum	
		Positive	Negative
BALF	Positive	19	89


	Negative	43	107




*BALF, bronchoalveolar lavage fluid.*

In terms of detecting resistance to the anti-TB drug rifampicin, all test results from culture and Xpert MTB/RIF were completely consistent with each other. The *kappa* value for the culture vs. Xpert MTB/RIF test results is 1.0 (**Table 5**), suggesting that Xpert MTB/RIF is a reliable method for the detection of antimicrobial resistance in TB.

**Table 5:** Agreement of rifampicin resistance test results between the Xpert MTB/RIF and culture methods.

		Culture	
		S	NS
Xpert MTB/RIF	S	85	0


	NS	0	3




*S, sensitive; NS, non-sensitive.*

### Comparisons of the Sensitivities of Parallel Tests

**FIGURE 3:**
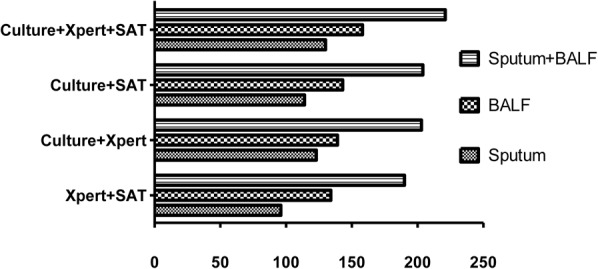
Positive rates of parallel tests using culture, Xpert MB/RIF, and SAT-TB in different specimen types. BALF, bronchoalveolar lavage fluid.

In the groups of only sputum and only BALF samples, a parallel tests model using all three tests (culture, Xpert MTB/RIF, and SAT-TB) had the highest sensitivity (sputum: 0.5039 and BALF: 0.6124). When using only two tests, culture plus Xpert MTB/RIF and culture plus SAT-TB had the higher sensitivities (**Figure 3** and **Table 6**).

**Table 6:** Comparison of the sensitivities and 95% confidence intervals of parallel tests with different sample types.

	Sputum	BALF	Sputum+BALF
Xpert MTB/RIF+SAT-TB	0.3721 (0.3154–0.4325)	0.5194 (0.4586–0.5796)	0.7364 (0.6795–0.7864)


Culture+Xpert MTB/RIF	0.4767 (0.4166–0.537)	0.5388 (0.4778–0.598)	0.7868 (0.7328–0.832)


Culture+SAT-TB	0.4419 (0.3826–0.5029)	0.5543 (0.4933–0.6137)	0.7907 (0.7370–0.8359)


Culture+Xpert MTB/RIF +SAT-TB	0.5039 (0.4433–0.5644)	0.6124 (0.5517–0.6698)	0.8566 (0.8086–0.8941)




*BALF, bronchoalveolar lavage fluid.*

### Comparisons of the Sensitivities of Joint Detections Using Sputum and BALF Samples

In the joint sample detection using both sputum and BALF samples, the combination of all three methods (culture, Xpert MTB/RIF, and SAT-TB) had the highest sensitivity (0.8566). The sensitivities in the models using only two tests ranged between 0.7364 and 0.7907. Overall, the sensitivities of the detection models using joint samples were significantly higher than those using a single sample (*p* < 0.05, **Table 6**). Relative data from Supplementary Table S1 was shown in Supplementary Data Sheet 2.

### Results of Patient Follow-Up

A total of 258 cases were diagnosed as active PTB based on the applied diagnostic criteria, and they all received anti-TB treatment. Of these, 221 cases of PTB (85.65%) were confirmed based on parallel tests using sputum and BALF samples. Patient follow-up revealed that all patients achieved a satisfactory response to anti-TB chemotherapy.

## Discussion

Laboratory test results are an important and definitive basis for TB diagnosis (Hu, 2008; Salina and Morozova, 2008; Melinte, 2009; Amaral and van Soolingen, 2015; Purohit and Mustafa, 2015; Purohit et al., 2015; Sharma et al., 2015; Shiferaw et al., 2015; Afsar and Afsar, 2016; Dunn et al., 2016; Procop, 2016; Shi et al., 2016; Bhirud et al., 2017). At present, the main clinical diagnostic methods for PTB are smear, culture, biophage, and molecular and immunological methods (Brent et al., 2011; Barnard et al., 2012; Li et al., 2015; Wang et al., 2015; Huo and Peng, 2016; Penata et al., 2016; Shi et al., 2016; Gelalcha et al., 2017; Maharjan et al., 2017; Yadav et al., 2017). However, in terms of their sensitivity, all single tests have defects of varying degrees in clinical practice.

Previously published meta-analysis literature has proven that some methods, such as culture and nucleic acid amplification tests, are relatively reliable tools for diagnosing PTB (Chang et al., 2012; Yuan et al., 2014; Kaur et al., 2016; Nagai et al., 2016; Li et al., 2017). Based on systematic review, the WHO now recommends nucleic acid amplification tests, such as Xpert-MTB/RIF, over conventional tests for the diagnosis of TB in lymph nodes and other tissues and as the preferred initial test for the diagnosis of TB meningitis (Denkinger et al., 2014). However, due to a lack of systematic review and meta-analysis on this specific use, the diagnostic value of Xpert-MTB/RIF is unclear for latent TB.

According to a meta-analysis of nine studies with a total of 1,214 subjects, nucleic acid amplification tests on BALF samples have important diagnostic value for smear-negative PTB, and the pooled sensitivity and specificity were 0.54 [95% confidence interval (CI): 0.48–0.59] and 0.97 (95% CI: 0.95–0.98), respectively (Tian et al., 2015). Based on these findings, the present study selected culture, Xpert MTB/RIF, and SAT-TB for use in the parallel detection of TB to potentially improve TB diagnostic performance.

According to the quantitative results from Xpert MTB/RIF, the MTB levels were very low to medium in the majority of patients in this study. The relatively low levels of MTB may have been the reason for the negative smear results in these patients. By applying parallel tests models using joint samples, we confirmed a total of 221 (85.65%) of the 258 enrolled PTB cases. According to the 6-month follow-up reports, all 258 patients had a good therapeutic response, suggesting that the diagnoses were reliable.

Overall, the sensitivities of single-test models using culture, Xpert MTB/RIF, or SAT-TB were lower than those of parallel test models, regardless of whether sputum specimens or BALF samples were used. However, we found that there was relatively low agreement between the detection results from sputum samples and those from BALF samples, and the sensitivity in BALF samples was higher than that in sputum samples for all three tests. Parallel tests models using culture plus Xpert MTB/RIF plus SAT-TB, culture plus Xpert, or culture plus SAT-TB achieved higher sensitivities for MTB detection in PTB. Furthermore, the joint detection using sputum and BALF samples can further increase the diagnostic sensitivity for smear-negative PTB.

The above results indicate that single test models have a low sensitivity; thus, we recommend that a parallel tests model of PTB diagnosis be considered in clinical practice. Some methods, such as culture, are excessively time-consuming (4 weeks), so applying a test model using Xpert MTB/RIF in combination with SAT-TB (2 h) as an alternative would greatly save time. Our results also confirm that Xpert MTB/RIF is reliable for the detection of rifampicin resistance. In terms of expense, the tests using culture, Xpert MTB/RIF, and SAT-TB cost $16, $64, and $16, respectively, culture takes 2 weeks while Xpert MTB/RIF and SAT-TB only take 2 h. Although Xpert MTB/RIF is relatively expensive than SAT-TB and culture, Xpert MTB/RIF can provide the result of rifampin resistant as well, these prices are generally affordable for most families From an applied point of view, bronchoscopy can be performed in many urban hospitals in China, and MTB culture and molecular diagnosis using BALF sample are feasible and appropriate for PTB diagnosis.

Given our findings, we recommend that culture and molecular biology diagnosis be conducted using sputum specimens for suspected PTB cases. A positive result of two molecular tests indicates that the clinician can make a definite diagnosis; however, if the results of molecular tests using sputum samples are negative, BALF specimens should be collected by bronchoscopy and used for molecular diagnosis and culture. In cases where patients are difficult to diagnose because of a lack of electronic bronchoscopy or laboratory conditions in the primary medical establishment, the patients should be transferred to a qualified setting for further diagnosis.

## Conclusion

Our data suggest that the application of parallel tests using culture, Xpert MTB/RIF, and SAT-TB in both sputum and BALF samples will significantly increase the diagnostic performance of smear-negative PTB. Therefore, this model should be applied in clinical settings where appropriate.

## Author Contributions

LF, CZ, and ZZ did the study design. LF and CZ wrote the manuscript. DL and JN did the statistic analysis. JG, HL, SZ, LY, and XH performed the bronchoscopy and collected the samples. LF and ZZ did the fund support.

## Conflict of Interest Statement

The authors declare that the research was conducted in the absence of any commercial or financial relationships that could be construed as a potential conflict of interest.
