# Supplementary material for: Parallel Tests Using Culture, Xpert MTB/RIF, and SAT-TB in Sputum Plus Bronchial Alveolar Lavage Fluid Significantly Increase Diagnostic Performance of Smear-Negative Pulmonary Tuberculosis
Source: Front Microbiol. 2018 Jun 15;9:1107. doi: 10.3389/fmicb.2018.01107 (PMC6020777; doi:10.3389/fmicb.2018.01107)
Supplement: Supplementary file 2 [file Data_Sheet_1.doc]

Supplementary data 2 (S2)

Table 1. Comparisons of the sensitivities of single test with 95% confidence interval in different samples.

|  | Sputum | BALF | Sputum +BALF |
| --- | --- | --- | --- |
| Culture | 91 | 101 | 162 |
| Xpert | 83 | 108 | 162 |
| SAT | 62 | 108 | 151 |

BALF : Bronchoalveolar lavage fluid.

Table 2 Agreement of test results of culture between sputum and BALF.

|  |  | Sputum | |
| --- | --- | --- | --- |
|  |  | Positive | Negative |
| BALF | Positive | 30 | 71 |
|  | Negative | 61 | 96 |

Table 3 Agreement of results of Xpert MTB/RIF between sputum and BALF.

|  |  | Sputum | |
| --- | --- | --- | --- |
|  |  | Positive | Negative |
| BALF | Positive | 29 | 79 |
|  | Negative | 54 | 96 |

BALF : Bronchoalveolar lavage fluid.

**Table 4 Agreement of results of SAT-TB between sputum and BALF.**

|  | Sputum | | |
| --- | --- | --- | --- |
|  |  | Positive | Negative |
| BALF | Positive | 19 | 89 |
|  | Negative | 43 | 107 |

BALF : Bronchoalveolar lavage fluid.

Table 5 Agreement of test results of rifampicin resistance between Xpert MTB/RIF and culture method.

|  |  | Culture | |
| --- | --- | --- | --- |
|  |  | S | NS |
| Xpert MTB/RIF | S | 85 | 0 |
|  | NS | 0 | 3 |

Table 6 Comparisons of the sensitivities of parallel tests with 95% confidence interval in different samples.

|  | Sputum | BALF | Sputum+BALF |
| --- | --- | --- | --- |
| Xpert+SAT | 96 | 134 | 190 |
| Culture+Xpert | 123 | 139 | 203 |
| Culture+SAT | 114 | 143 | 204 |
| Culture+Xpert+SAT | 130 | 158 | 221 |

BALF : Bronchoalveolar lavage fluid.
